# Study of Functional and Allosteric Sites in Protein Superfamilies

**Published:** 2015

**Authors:** D. Suplatov, V. Švedas

**Affiliations:** Lomonosov Moscow State University, Belozersky Institute of Physicochemical Biology, Vorobjev hills 1-40, Moscow 119991, Russia; Lomonosov Moscow State University, Faculty of Bioengineering and Bioinformatics, Vorobjev hills 1 -73, 119991, Moscow, Russia

**Keywords:** binding sites, catalytic site, allosteric site, function, regulation, bbb, structure-function relationship, bioinformatics

## Abstract

The interaction of proteins (enzymes) with a variety of low-molecular-weight compounds, as well as protein-protein interactions, is the most important factor in the regulation of their functional properties. To date, research effort has routinely focused on studying ligand binding to the functional sites of proteins (active sites of enzymes), whereas the molecular mechanisms of allosteric regulation, as well as binding to other pockets and cavities in protein structures, remained poorly understood. Recent studies have shown that allostery may be an intrinsic property of virtually all proteins. Novel approaches are needed to systematically analyze the architecture and role of various binding sites and establish the relationship between structure, function, and regulation. Computational biology, bioinformatics, and molecular modeling can be used to search for new regulatory centers, characterize their structural peculiarities, as well as compare different pockets in homologous proteins, study the molecular mechanisms of allostery, and understand the communication between topologically independent binding sites in protein structures. The establishment of an evolutionary relationship between different binding centers within protein superfamilies and the discovery of new functional and allosteric (regulatory) sites using computational approaches can improve our understanding of the structure-function relationship in proteins and provide new opportunities for drug design and enzyme engineering.

## INTRODUCTION

Understanding the relationship between protein structure and function is one of the most challenging problems of modern biochemistry. It is complicated due to the fact that similarity of structures does not imply a common function – proteins with different properties can share a common structural framework [1, 2], while the same function can be performed by proteins with different folds [3]. Specific protein-protein interactions and recognition of low-molecular-weight compounds are crucial to all living systems. To understand the molecular mechanisms of these processes and the structure- function relationship in proteins, it is necessary to study the structural organization of the specific sites responsible for the binding of various ligands (substrates, inhibitors, effectors) [4]. Analysis and functional classification of pockets and cavities on the protein surface which form binding sites with unique properties can lead to a better understanding of the molecular mechanisms of protein functions, facilitate function prediction of recently discovered enzymes, and provide new opportunities for protein/enzyme engineering and drug design.

**Fig. 1:**
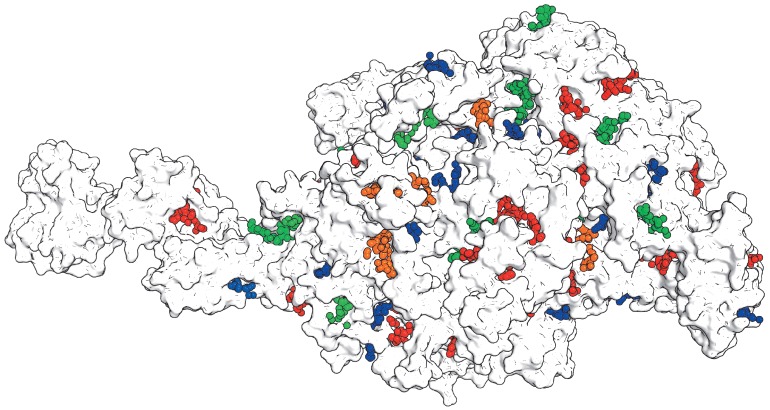
Potential binding sites for low-molecular-weight effectors in the structure of bacterial RNAP. Clusters of same-colored spheres mark the potential sites on the protein surface and represent centers of α-spheres that fill in the volume of the corresponding binding pocket (see Appendix). The figure was prepared using PyMol based on the crystal structure 1YNN from PDB

When protein function is investigated the functional sites – active sites of enzymes, channels of membrane transport proteins, DNA- and protein-binding motifs of different regulatory proteins – attract the most attention. However, during recent years we have witnessed the increasing role of the allostery phenomenon – regulation of protein functions at the binding of low-molecular-weight effectors in regulatory sites which are topologically independent of functional sites [5]. These facts have stimulated research activities to understand the regulation of the biological macromolecules’ function caused by interaction with different ligands in allosteric centers. Several experimental and computational approaches have been developed to search for new regulatory sites in protein structures. Attempts have been made to understand the relationship between the functional and regulatory centers located at a considerable distance from each other and explore the molecular mechanisms of their interaction [6]. Low-molecular-weight inhibitors have been discovered that are capable of selective interaction with allosteric sites in various proteins associated with human diseases [7]. However, the particular attention to this issue is not so much because of the unique features of specific proteins, but is rather due to the general importance of these processes for functional regulation in living organisms. There are reasons to believe that allostery is a universal phenomenon common to most proteins [8], which in addition to our interest into fundamental mechanisms draws attention due to its potential applications in biotechnology and biomedicine. Recent studies have shown that proteins and enzymes, along with quite well-studied functional sites (active sites) and allosteric centers, contain a significant amount of virtually unexplored potential binding pockets. *Fig. 1* shows the structure of DNA-dependent RNA polymerase – a key enzyme of RNA synthesis in all living organisms [9, 10]. The surface of this large multi-subunit protein is covered by a large number of cavities – potential binding sites. These include the active center containing catalytic residues and the DNA binding motifs, as well as several known allosteric sites capable of binding a variety of low-molecular-weight ligands [11, 12]. The role of other binding sites, i.e. the majority of existing pockets in this case, remains unknown. How important are these sites for enzyme function? Which binding centers play a physiological role and which can be used to create a protein with new properties for practical applications? How to evaluate the potential role of each specific site for the regulation of protein function?

In this review, we discuss the study of the structure- function relationship in proteins based on the analysis of various binding sites in their structures. In this context, experimental and computational approaches are considered which allow us to search for new binding centers capable of interacting with regulatory ligands and study the molecular mechanisms of allostery and the relationship between function and regulation in proteins. The rapid expansion of public databases makes genomic, structural, and functional information widely available for a large number of proteins. In this respect, bioinformatic methods provide an opportunity to study protein functions within the corresponding superfamilies systematically, rather than individually. Analysis of the structural information and experimental data concerning individual proteins, as well as in their relationship with close and distant evolutionary relatives, should contribute to a better understanding of the structure-function relationship in proteins/enzymes and unveil new mechanisms of regulation of their functional properties.

## THE PHENOMENON OF ALLOSTERY

Allostery is generally defined as the process of regulation of protein function due to the binding of an effector – a ligand or another protein – in a site on the protein surface referred to as an allosteric center [6]. The term “allosteric” comes from the Greek roots *allos* (other) and *stereos *(solid), and can be translated as “different shape” in order to emphasize the relationship of conformational states between structurally remote sites in proteins [8]. It is known that allosteric regulation of metabolism is important for all living cells, and allosteric effectors can be either inhibitors or activators with respect to protein function [13].

**Fig. 2:**
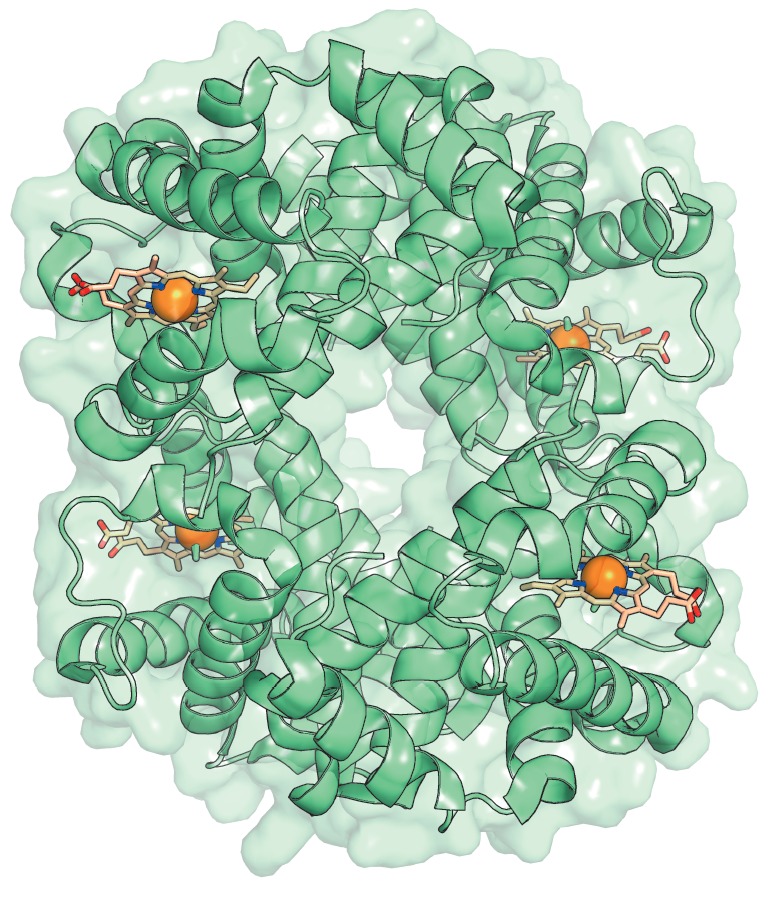
Three-dimensional structure of human hemoglobin. Heme molecules (orange) are shown as sticks in each subunit of the tetramer. The figure was prepared using PyMol based on the crystal structure 1GZX from PDB

Historically, allostery has typically referred to a cooperative effect in multi-subunit proteins that function at the quaternary structural level. The first “concerted” molecular model of allostery, known as the MWC (Monod-Wyman-Changeux) model, was proposed in 1965 based on the 24 then known examples [14]. The abrupt increase in hemoglobin oxygen affinity described by an S-shaped curve suggested a cooperative effect. However, a crystal structure of hemoglobin obtained in 1960 with 5.5A resolution showed that heme molecules that bind oxygen are located in different subunits of the protein at a considerable distance from each other (*Fig. 2*) [15]. This led to the conclusion that allosteric proteins have a symmetrical arrangement of subunits which can adopt at least two conformational states – R (relaxed) or T (tense), characterized, in the case of hemoglobin, by high and low affinity for oxygen, respectively. The transition from one conformational state to another as a result of ligand binding proceeds in a coordinated manner among all subunits so that the oligomeric protein does not exist in a hybrid state RT. This simplified model was used to kinetically characterize hemoglobin saturation by oxygen [14]. However, the molecular mechanism of this phenomenon remained unclear until a series of structural studies [16, 17]. It was shown that the binding (release) of oxygen is accompanied by significant changes in the spatial organization of the functional center and disruption (formation) of a few salt bridges. This leads to displacement of subunits relative to one another so that binding of the first oxygen molecule affects the whole tetramer. In other words, binding (release) of one molecule of the substrate to one subunit changes oxygen affinity in other subunits, making hemoglobin an effective oxygen carrier along the pressure gradient. Such cooperative effects in homo-oligomeric proteins/enzymes are one of the most known examples of allostery. In this case, the active center of one subunit can function as an allosteric center with respect to the active center of another subunit. Therefore, the binding of the second substrate molecule (or the corresponding analogue) may not be accompanied by its catalytic conversion but leads to the allosteric effect on the binding site of the first substrate molecule. According to the “sequential” or KNF model (Koshland-Nemethy-Filmer), subunits within the multimer change their conformation one at a time; i.e. binding of a ligand changes the conformation and properties of the corresponding subunit and affects its neighbors [18]. In other words, ligand binding causes consecutive conformational changes in protein subunits; e.g., this model describes negative cooperativity in the enzyme glyceraldehyde-3-phosphate dehydrogenase. Binding of a coenzyme NAD^+^ to the active site of one subunit weakens its binding to the adjacent subunit due to the rearrangement of intra- and inter-subunit contacts [19, 20]. This property maintains enzyme activity at a constant level regardless of the concentration of a ligand in the environment. Although an attempt was made to combine the MWC and KNF models into a more general one [21], further studies showed that molecular mechanisms of allosteric regulation are so complex and diverse that none of the proposed simplified models can exhaustively describe the phenomenon of allostery.

Nowadays, it is nearly generally accepted that not just multi-subunit proteins, but also monomeric ones are subjected to allosteric regulation, and allosteric ligands are mainly considered as low-molecular-weight compounds that bind to regulatory sites topologically independent of functional centers. In addition, the regulatory effects caused by protein-protein interactions, phosphorylation, and even point mutations are sometimes also considered as allosteric ones. The diversity of allosteric mechanisms in various proteins and enzymes is well illustrated in recent publications [5, 7, 22]. It has been suggested that allostery may be an intrinsic property of virtually all proteins [8]. The exceptions, probably, draw up structural proteins with rigid conformations that limit their flexibility and opportunities for regulation. Indeed, there is growing experimental evidence of allostery in enzymes that were previously considered as non-allosteric.

Phosphofructokinase catalyzes one of the key steps in glycolysis and offers an example of a protein whose function can be regulated by various effectors. Allostery has been described in this superfamily for enzymes from both prokaryotes [23] and eukaryotes, the latter being characterized by much larger globules due to duplications, insertions, and mutations of the ancestral prokaryotic gene, which contributed to the emergence of new allosteric centers [24]. At the same time, phosphofructokinase from fungus *Dictyostelium discoideum* is different from its homologs and considered as non-allosteric. However, it was shown that deletion of one C-terminal leucine residue leads to the emergence of allosteric properties in this enzyme similar to other superfamily members [25]. A different example is allosteric regulation in pyruvate kinases [26]. Four isoforms of this enzyme have been characterized in mammalian tissues – L, R, M_1_, and M_2_. All isoforms, except for M1, are allosteric enzymes and show positive homotropic cooperativity with respect to the substrate, as well as positive heterotropic cooperativity with respect to fructose-1,6-bisphosphate. Isoforms M_1_ and M_2_ were shown to be produced from a common gene by alternative splicing. The corresponding primary sequences are different in 23 amino acid residues which are located at the intersubunit interface and are involved in the formation of the binding site of fructose-1,6-bisphosphate. It has been shown that two point mutations introduced into the structure of M_1_ isoform – at the intersubunit interface [27] and in the binding site of fructose-1,6-bisphosphate [26] – lead to the emergence of allosteric properties similar to those of other homologs. In a different study, myoglobin, a paralog1 of hemoglobin, has been shown to exist in three major conformational states with different catalytic properties – the so-called taxonomic substates – and each of these assumes a very large number of slightly different conformations or statistical substates [28]. Based on this observation, it has been further assumed that bimolecular reactions with diatomic molecules (e.g., NO and O_2_) can be allosterically controlled in myoglobin due to changes in the geometry of conservative cavities adjacent to the active center. It is interesting to note that in all these cases allosteric regulation has been discovered in proteins which are evolutionary related to other allosteric enzymes. These examples speak not only to the wide occurrence of allostery, but also underline the general mechanisms of this phenomenon within protein superfamilies. They indicate the possibility of fine-tuning allostery by only several point mutations, but also emphasize the complex relationship between function and regulation.

The current concept of protein structure assumes that proteins exist as complex statistical ensembles of conformers that fold and unfold continuously by making local rearrangements [6, 29]. In this context, the allosteric effect is a result of the redistribution of conformational states [8]. In other words, binding of an allosteric effector leads to a population shift toward conformational states that are significantly different in functional terms from the native state [30]. On the other hand, if a protein is considered to be non-allosteric, this can simply be an indication that alternative conformations of binding sites and functionally important conformational transitions have not been discovered yet. It does not mean, however, that one cannot choose a ligand or specific environmental conditions that would be able to cause a conformational redistribution and trigger allosteric behavior in otherwise not-allosteric proteins. In principle, almost any substance bound to the protein surface can cause population shift of conformational states, the question being only in the effectiveness of the shift and its impact on protein function [8]. Further studies with various proteins should be performed to understand this problem. However, the conformational changes associated with allosteric regulation are difficult to detect using current experimental techniques. The recently developed bioinformatic and computational biology approaches provide new opportunities for solving this problem.

## IDENTIFICATION OF BINDING SITES IN PROTEIN STRUCTURES

The prediction of binding sites in proteins based on information about their structures is a new challenging field in computational biology [31]. Various geometry-based structural approaches to the search for pockets and cavities on the protein surface have been developed (*Table*). Often, several pockets are found in a protein structure and an attempt is further made to select the most relevant sites that are likely to bind a ligand – by implementing various geometric criteria (size, depth, and orientation of a potential binding cavity [32-34]) or a statistical analysis that takes into account the physicochemical properties of the known ligand binding sites [35, 36]. Alternatively, energy-based approaches have been proposed that predict and rank binding pockets by calculating the binding energy of small organic molecules (probes) on the protein surface [37, 38]. All these approaches basing on the analysis of the available protein structure can quickly and efficiently detect cavities and pockets that form potential binding sites, but they give no idea about their functional significance and structure of the complementary ligands.

**Table:** Online services to predict binding sites in protein structures and rank them by functional significance

Name	On-line address	Algorithm used to identify the binding sites	Algorithm used to rank the binding sites
Fpocket [35]	http://mobyle.rpbs.univ-paris-diderot.fr/ - Programs - Structure - Pockets - fpocket	Geometric, based on Voronoi tessellation and detection of α-spheres	Statistical, by estimating similarity to known ligand binding sites
POCASA [33]	http://altair.sci.hokudai.ac.jp/g6/service/pocasa/	Geometric, by rolling spherical probes along the protein surface	Geometric, taking into account the position and size of the pocket
pocketZebra [39]	http://biokinet.belozersky.msu.ru/pocketzebra	Geometric, based on Voronoi tessellation and detection of α-spheres	Bioinformatic, analysis of subfamily-specific positions in protein superfamilies
SiteHound [38]	http://scbx.mssm.edu/sitehound/sitehound-web/Input.html	Energy-based, by estimating the interaction energy of amino acids at the protein surface with carbon or phosphate chemical probes	Energy-based, by estimating the interaction energy of amino acids at the protein surface with carbon or phosphate chemical probes
LIGSITEcsc [41]	http://projects.biotec.tu-dresden.de/pocket/	Geometric, based on the calculation of the Connolly surface	Bioinformatic, analysis of conserved positions

In the course of evolution of proteins from a common ancestor, some functional properties were preserved, while others underwent changes as a result of natural selection, which led to functional diversity. For example, homologous enzymes within a superfamily can share a common fold and reaction chemistry but differ in other functional properties (e.g., substrate specificity, enantio- and regio-selectivity, and type of catalyzed chemical transformation), as well as principles of their regulation. The continuous growth in public databases providing access to genomic and structural information on various proteins and enzymes opens new perspectives for a large-scale comparative analysis of both evolutionarily close and distant relatives within protein superfamilies. Not all positions in protein structures are equally susceptible to variation in the course of evolution, reflecting differing selection pressure on amino acids residues with different functional roles. That makes it possible to apply a bioinformatic analysis of protein superfamilies to the study of the evolutionary relationship of amino acid residues in functional and regulatory binding sites [39] (*Table*). Totally conserved positions play a key role in a function common to all proteins within a superfamily; e.g., they are involved in the enzyme’s catalytic mechanism. It should be noted, however, that catalytically important amino acids are not always conserved throughout enzyme superfamilies and can even migrate within a common structural framework of homologous proteins [2, 40]. Catalytic nucleophile in α/β-hydrolases can be represented by serine, cysteine or aspartate, and the catalytic acid can be located in at least two alternative positions of the main polypeptide chain. Nevertheless, it was shown that conservation of residues in pockets and cavities in the protein structure is an efficient criterion for annotation of functional centers [41-43] and can be used to characterize a wide range of enzymes [44-46]. In fact, when characterizing a new protein with an unknown function it is reasonable to begin with a comparative analysis of its closest homologs in order to identify the conserved positions in columns of the corresponding multiple alignment. The role of the most conserved residues can be further studied experimentally by introducing point mutations and evaluating their impact on the protein function or enzyme catalytic properties. Annotation of functional sites in a new protein by residue conservation can be performed even in the absence of structural data given that the appropriate information is available for evolutionary relatives. This type of data for various proteins from different superfamilies is in constant growth and is being accumulated in public databases (see next chapter). To sum up, integration of geometry-based structural methods with bioinformatics approaches can provide more efficient annotation of functional centers in proteins.

**Fig. 3:**
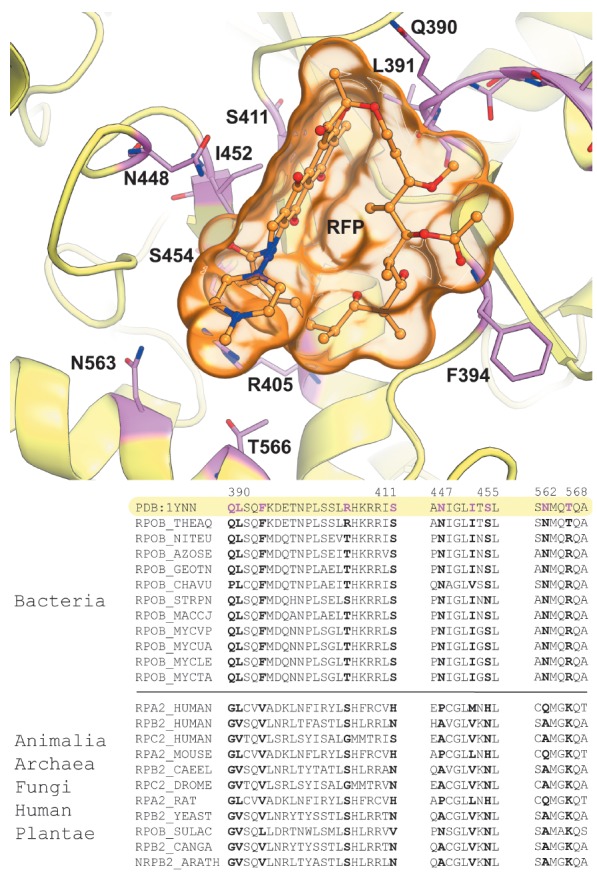
Binding of rifampicin (RFP) to the allosteric site in β-subunit of bacterial RNAP. Sticks represent subfamily-specific positions (pink) identified by the bioinformatic analysis of 271 RNAPs from different sources, and corresponding fragments of the multiple alignment are shown. An interactive version of this illustration is available online (see Appendix). The figure was prepared using PyMol based on the crystal structure 1YNN from PDB

A comparative study of various proteins allowed researchers to conclude that allosteric sites in enzyme superfamilies are characterized by a lower content of conserved positions and a higher content of variable positions [47]. It has been further shown that mutagenesis of variable positions in allosteric centers leads to a change in the allosteric effect, while substitution of conserved positions in these centers leads, as a rule, to a loss of catalytic function. These results demonstrate that residue conservation may not be a suitable criterion to annotate regulatory centers but indicate the important role of variable positions in the binding of ligands and allosteric regulation of functional properties in proteins superfamilies. In this regard, the subfamily- specific positions (SSPs) – conserved within functional subfamilies, but different between them – attract special attention [48, 49]. SSPs are observed in both catalytic and allosteric sites, and their presence can be a very powerful factor for the identification of functional and regulatory centers in protein structures [39]. Identification of statistically significant subfamily- specific positions can help understand the difference in the organization of binding sites within evolutionarily related proteins. DNA-dependent RNA polymerase (RNAP) is a key enzyme in DNA transcription crucial to all living systems. The catalytic core of the bacterial enzyme consists of subunits α2ββ’ω, which are characterized by a high degree of structural and functional similarity among homologs from different organisms. Bacterial RNAP is also a confirmed target for antimicrobial drugs [12]. The first-line anti-tuberculosis drug rifampicin selectively inhibits transcription in *Mycobacterium tuberculosis *due to interaction with the allosteric center located in the β-subunit of this enzyme. Interaction of the inhibitor with the bacterial enzyme directly blocks the elongation path in the pathogen, without affecting the homologous human enzyme [50]. Bioinformatic analysis of the RNAP superfamily shows that the selective rifampicin binding by the bacterial enzyme is caused by the presence of different amino acid residues in prokaryotic and human proteins at the subfamily-specific positions of the corresponding binding site (*Fig. 3*). This example shows that the role of SSPs in binding sites which interact with regulatory ligands should be further evaluated to better understand the molecular mechanisms of specific recognition of allosteric effectors and reveal patterns of functional regulation in protein superfamilies.

## PUBLIC DATABASES

The speed and availability of versatile information from open sources through the Internet is an important driving force in modern science. In this context, a special role belongs to the numerous public databases.

The Catalytic Site Atlas (CSA) database is one of the main resources related to enzymes [51]. The core of CSA is the experimental data on 1,000 catalytically active proteins with different properties, and bioinformatic methods are further implemented to search for similar amino acid sequences and accurately annotate catalytic residues basing on their conservation patterns. As a result, the database provides information about tens of thousands of proteins.

Information relating to allosteric proteins is not as abundant due to the lack of corresponding structural data and difficulties in determining the allosteric sites; however, during the last decade progress has been achieved in this area. Compared to the 24 allosteric proteins discovered more than 50 years ago (when the first model of cooperativity was proposed), today hundreds of documented cases are reported. The recently founded Allosteric Database (ASD) has been the first attempt to generalize relevant data from the literature [52]. Today ASD contains nearly 2,000 sites. However, not all entries are supported by structural data about a protein complex with an effector, and some annotations seem controversial. Nevertheless, it shall be expected that the systematization of experimental data related to the structure and function of allosteric centers will be continued, also in other public databases.

## THE RELATIONSHIP BETWEEN FUNCTION AND REGULATION

There is no doubt that a conformational change in protein structure caused by binding of a ligand to an allosteric center eventually leads to a change in its functional properties. However, little is known about the particular molecular mechanisms of this phenomenon. How to explain the observed cooperativity at binding of various ligands and how to predict the relationship between independent sites in proteins which are not ascribed yet as allosteric ones? Several attempts have been undertaken during recent years to understand the relationship between function and regulation [6]. These studies were aimed at a computational search for correlations – structural or evolutionary – between events occurring in topologically independent centers on the protein surface upon binding of ligands. Let us consider some such examples.

**Fig. 4:**
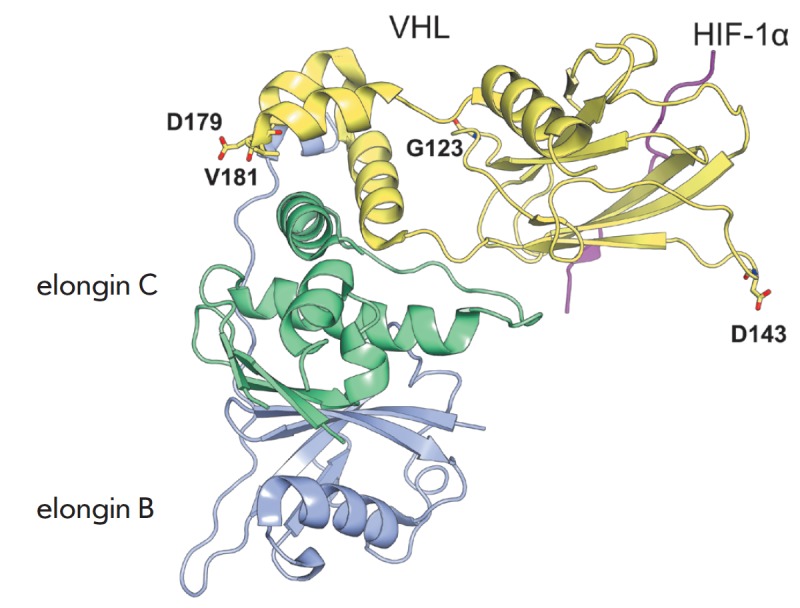
The complex of the transcription factor HIF (magenta) – von Hippel-Lindau tumor suppressor protein pVHL (yellow) – elongin C (green) – elongin B (blue). The α domain of pVHL interacts with elongin C, while the β domain binds HIF. Amino acid residues in pVHL whose motions were strongly correlated with the unstable inter-domain region are shown as sticks. The figure was prepared using PyMol based on the crystal structure 1LM8 from PDB to illustrate the results of [56]

Structural changes that occur as a result of ligand binding are directly related to the conformational mobility of the protein globule. Molecular dynamics has proven efficient in studying structural changes in proteins [53, 54], including correlated fluctuations of atoms occurring as a result of collective movement [55]. Covariance maps of atomic fluctuations along MD trajectories have been calculated and used to study the molecular mechanisms of allosteric regulation in the von Hippel-Lindau tumor suppressor protein (pVHL) [56]. Free pVHL is only marginally stable and exists in the so-called “molten globule” state. pVHL stability is greatly improved after binding to elongin C and elongin B, and these proteins together form a substrate-recognition component for the hypoxia-inducible factor (HIF) within the E3 ubiquitin protein ligase complex (*Fig. 4*). MD study has shown that the interface between the pVHL α and β domains is the most unstable region of the protein. Amino acid residues in pVHL have been selected whose motions were strongly correlated with this unstable motif. Molecular modeling has shown that introduction of amino acids from a more stable *Caenorhabditis elegans *pVHL into human pVHL at the selected positions results in significant stabilization of the protein in both the free state and within the complex. In other words, mutation of pVHL residues which are located away from elongin C and HIF binding sites has led to stabilization of the pVHL-elongin C complex and lowered the binding free energy of pVHL with HIF. The authors of [56] conclude that the stability and efficiency of binding to pVHL could be regulated allosterically by drugs mimicking the effect of the introduced mutations.

**Fig. 5:**
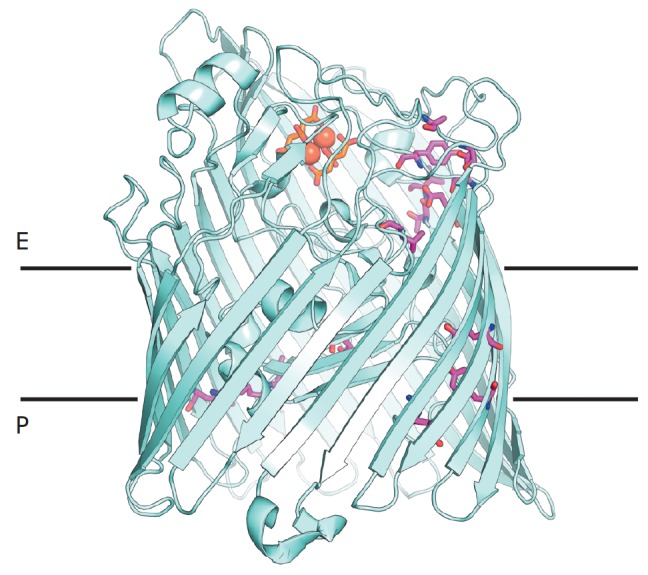
The structure of the outer membrane transport protein FecA. The relative position of the periplasmic surface (P) and extracellular surface (E) is shown. Ferric ions solubilized by ferric citrate (orange) are shown in the siderophore binding site. Magenta sticks correspond to the correlated residues involved in the formation of a network of interactions that provide functional communication between the periplasmic and extracellular binding sites in FecA. The figure was prepared using PyMol based on the crystal structure 1KMP from PDB to illustrate the results of [59]

A different example demonstrates the value of evolutionary correlations obtained from a statistical analysis of genomic sequences [57, 58]. The approach is based on the assumption that if two sites in a protein structure are functionally related, then the corresponding amino acid residues in homologous proteins should have been coevolving during evolution from a common ancestor, and therefore this correlation can be detected by a statistical comparison of amino acid sequences. In such a case, the correlation of amino acid occurrence at two sites in structures of related proteins can indicate the existence of a functional dependence between them. This approach has been used to analyze membrane protein FecA – a member of the TonB-dependent transporters family whose function is to pump iron through the outer membrane into the cells of gram-negative bacteria [59]. The interaction of a periplasmic domain of the TonB protein, which is involved in maintaining the proton gradient across the cytoplasmic membrane, with a conserved N-terminal TonB-binding motif of the transporter is an essential step of iron transport. It was suggested that TonB binding at the periplasmic surface is somehow dependent on a siderophore binding at the extracellular surface and causes conformational changes in the transporter protein that drive iron import. *Siderophores – low-molecular-weight compounds with high affinity to ferric ions (e.g., ferric citrate).* However, the specific mechanisms of the allosteric communication between the two binding sites located in different cell compartments at a considerable distance from each other are unknown. Statistical sequence analysis of TonB-dependent transport proteins revealed a sparse, but structurally connected network of evolutionarily correlated residues which provide functional communication between the periplasmic and extracellular binding sites in FecA (*Fig. 5*). Mutation of the selected residues, which were not directly involved in binding of either TonB or siderophore, has led to the disruption of FecA transport function.

To sum up, the available results suggest that identification of evolutionary and structural correlations presents an important tool to study the molecular mechanisms of allosteric regulation in proteins.

## BINDING SITES IN BIOTECHNOLOGY AND BIOMEDICINE

Binding sites of substrates/ligands in enzymes/proteins have been extensively studied to create new biocatalysts for industrial use (e.g., see review [60]), as well as inhibitors to treat human diseases [61, 62]. Although the choice of particular methods is specific to each case, the core principle of the most successful research projects can be described as “stochastic analysis.” The stochastic techniques, which are usually referred to as Directed evolution approaches, have been developed to produce enzymes with improved functional properties [63, 64]. These methods mimic the Darwinian process by combining random mutagenesis with screening and selection of the desired phenotype. Mutations are randomly introduced into the whole protein structure or in particular regions, and then their effect is evaluated experimentally to select the most promising substitutions which lead to an improvement in the desired properties. The stochastic approaches got much faster during the last decades due to implementation of the statistical analysis and computational tools [65, 66]. However, they remain resource-demanding, still require large mutant libraries, efficient screening techniques, and yet are able to scan only a small part of the sequence space. To sum up, random evolutionary methods are hampered by a high frequency of deleterious mutations and a low frequency of beneficial mutations. Similarly, the design of new drugs is usually based on a blind experimental screening of huge libraries of low-molecular-weight compounds in an attempt to find potential inhibitors of a target protein [67, 68]. Although the structure of the lead discovered by chance can be further optimized using experimental and computational methods, this approach in general is very resource-consuming and inefficient. Embracing this approach in 1995–2001, GlaxoSmithKline performed 70 high-performance experimental screening campaigns (US$1 million each) of selected target proteins from different pathogenic bacteria using original collections of potential inhibitors (which consisted of 260, 000–530, 000 compounds). Only 5 leads were found after seven years of research [69]. A review of the literature shows that similar studies were conducted between 1996 and 2004 by at least 34 different companies on 60 targets and are generally considered to have been unsuccessful [70]. The high costs and poor performance have eventually dampened interest in this empirical methodology.

Despite the apparent multipurposeness of stochastic approaches, they are usually aimed at studying the functional centers in proteins. To alter the catalytic properties of an enzyme random mutations are introduced into the structure of the active site [71]. Similarly, the majority of the developed drugs bind to functional centers of proteins (see [72] as an example). Practical application of allosteric sites is arguably rare, although some examples are known. It has been shown that introduction of a single mutation in the structure of glyceraldehyde-3-phosphate dehydrogenase leads to the destruction of a salt bridge near the active site and consequent loss of cooperativity in the binding of NAD^+^ [73]. The corresponding enzyme variant has been characterized by a two-fold increase in specific activity. Certain drugs are known that interact with the regulatory sites of proteins. Rifampicin and Myxopyronin bind to pockets within the β and β’ subunits of RNAP, which are topologically independent from the active site, and block enzyme operation [12]. Binding of doramapimod at the allosteric center of human p38 MAP kinase and consequent conformational rearrangements impose steric hindrance on the ATP binding [74]. Inhibitors of HIV-1 reverse transcriptase – efavirenz, nevirapine and delavirdine – bind to an allosteric site at a significant distance from the active site [75]. Summing up the existing experience, it should be noted that due to the higher sequence variability of allosteric sites within superfamilies these regulatory centers should be considered as no less attractive targets for selective inhibition than the catalytic sites [76].

The low efficiency of stochastic methods has stimulated the development of computational approaches to rationally design effective biocatalysts and find selective inhibitors of key metabolic enzymes. It has been shown that a bioinformatic analysis of the evolutionary relationships in functionally diverse protein superfamilies can be used not just to detect the key “hotspots” in enzyme structures, but also determine the specific amino acid substitutions to produce mutants with improved properties [54, 77-79]. Use of computational approaches in protein design has been recently reviewed [80-82]. The whole-genome sequencing of bacterial pathogens, including *Mycobacterium tuberculosis *[83], marked the beginning of computer genomics in medicine. Genomic approaches can be used to make a list of all target proteins in a particular organism and to identify the most promising ones for further experimental evaluation [84]. An important advantage of the post-genomic analysis is the ability to select taxonomically widely distributed molecular targets, as well as the ones specific to a particular organism. It was assumed that in this way one could create drugs with a broad therapeutic activity, as well as with high specificity to a particular pathogen. In addition, a comparative genomic analysis of bacteria and animals can be used to exclude proteins which have human homologs from the list of potential molecular targets. In such a way, it could be possible to avoid the toxicity of the drug [85]. It should be noted, however, that currently used postgenomic methods in drug discovery do not get into too much details when choosing molecular targets for new antibiotics; e.g., the first choice at selection of targets are proteins conserved in bacteria and absent from the human organism. The structural peculiarities of these proteins and the architecture of their binding sites are frequently left out of consideration. In general, exclusion from the list of potential molecular targets of those proteins of bacterial pathogens that have human homologs (in order to avoid the toxicity of the designed inhibitors) should be considered as quite unreasonable. The major metabolic pathways are mostly conserved, and the corresponding key enzymes are present in both pathogenic bacteria and man; e.g., we have already mentioned the first-line anti-tuberculousis drug rifampicin, which inhibits replication due to selective binding to the β-subunit of RNAP in bacteria, although the enzyme has a human homolog [50].

In summary, we can note the general trend away from inefficient stochastic approaches towards more rational and focused strategies. In this context, the role of bioinformatics and molecular modeling in biotechnology and biomedicine continues to grow steadily. Development of new approaches for a systematic analysis of various binding sites in large protein superfamilies will help, on the one hand, to establish a relationship between structure, function, and regulation of proteins/ enzymes, and, on the other hand, to detect binding sites for new substrates and inhibitors/effectors with a previously unknown mechanism of action.

## CONCLUSIONS

Protein-protein interactions, as well as interaction of proteins (enzymes) with a variety of low-molecular- weight compounds, are a crucial factor in the regulation of their functional properties. To date, research efforts have typically focused on studying ligand binding to the functional sites of proteins (active sites of enzymes), whereas the molecular mechanisms of allosteric regulation, as well as binding to other pockets and cavities in protein structures, remained poorly understood. In this context, it is of great interest not only to study the interaction between functional and allosteric centers, but also to identify and characterize new binding sites and their role in the regulation of protein function. Despite the first steps being made towards a better understanding of the relationship between structure, function, and regulation, the issue remains far from resolved and requires continued attention. Analysis of the available literature allows one to conclude that the role of bioinformatic methods and molecular modeling in investigating the role of different binding centers in protein function, as well as allosteric effects, will continue to grow. Establishment of an evolutionary relationship between different binding sites within protein superfamilies and discovery of new functional and allosteric (regulatory) sites using computational approaches will improve our understanding of the structure-function relationship in proteins and provide opportunities for creating new drugs and designing more effective biocatalysts.

## APPENDIX

**Multiple alignment of the RNAP superfamily of proteins**

The structure of bacterial RNAP (PDB code: 1YNN) was used as a seed. To reconstruct evolutionarily dis tant RNAPs from various organisms (bacteria, animal, human, etc.) structural similarity search versus PDB database was carried out using the superpose algorithm from the CCP4 package [86]. The lower bound for matching of secondary structure elements was set to 30% in each protein. For each detected remote homolog, a list of evolutionarily close proteins was reconstructed using the BLAST algorithm versus the Swiss-Prot database [87]. The resulting samples were filtered to remove redundant sequences at a 95% identity upper bound, as well as outliers with similarity of less than 0.5 bit score per column [88] compared to the respective homolog with a known structure. Structural alignments were carried out by the Matt algorithm [89], and sequence alignments were performed using T-coffee [90]. The resulting structural alignment of distant homologs was used as a core to align samples of closely related sequences. The resulting structure-guided multiple alignment of the RNAP superfamily contains 271 protein sequences.

**Binding site prediction**

Identification of pockets and cavities in the RNAP structure (code in PDB: 1YNN), which are potentially capable of binding small molecules, was performed by the Fpocket algorithm [35].

**Bioinformatic analysis**

Identification of the subfamily-specific positions and subfamily-specific binding sites in the RNAP superfamily was performed using the Zebra [91] and pocket- Zebra [39] algorithms.

**Structural analysis**

Visualization and analysis of protein structural information was performed using PyMol (Schrodinger LLC).

**Dissemination**

Online access to the results of the bioinformatics analysis of the superfamily of DNA-dependent RNA polymerases is provided at http://biokinet.belozersky.msu.ru/pocketzebra (see “Examples”).

## Abbreviations

PDB: Protein Data Bank
RNAP: DNA-dependent RNA polymerase
MD: Molecular Dynamics
NAD: nicotinamide adenine dinucleotide
SSP: subfamily-specific position
